# DUNDRUM-2: Prospective validation of a structured professional judgment instrument assessing priority for admission from the waiting list for a forensic mental health hospital

**DOI:** 10.1186/1756-0500-4-230

**Published:** 2011-07-03

**Authors:** Grainne Flynn, Conor O'Neill, Harry G Kennedy

**Affiliations:** 1 National Forensic Mental Health Service, Central Mental Hospital, Dundrum, Dublin 14, Ireland; 2 Department of Psychiatry, Trinity College, Dublin, Ireland

**Keywords:** waiting lists, triage, urgency, forensic psychiatry, secure hospitals, needs assessment

## Abstract

**Background:**

The criteria for deciding who should be admitted first from a waiting list to a forensic secure hospital are not necessarily the same as those for assessing need. Criteria were drafted qualitatively and tested in a prospective 'real life' observational study over a 6-month period.

**Methods:**

A researcher rated all those presented at the weekly referrals meeting using the DUNDRUM-1 triage security scale and the DUNDRUM-2 triage urgency scale. The key outcome measure was whether or not the individual was admitted.

**Results:**

Inter-rater reliability and internal consistency for the DUNDRUM-2 were acceptable. The DUNDRUM-1 triage security score and the DUNDRUM-2 triage urgency score correlated r = 0.663. At the time of admission, after a mean of 23.9 (SD35.9) days on the waiting list, those admitted had higher scores on the DUNDRUM-2 triage urgency scale than those not admitted, with no significant difference between locations (remand or sentenced prisoners, less secure hospitals) at the time of admission. Those admitted also had higher DUNDRUM-1 triage security scores. At baseline the receiver operating characteristic area under the curve for a combined score was the best predictor of admission while at the time of admission the DUNDRUM-2 triage urgency score had the largest AUC (0.912, 95% CI 0.838 to 0.986).

**Conclusions:**

The triage urgency items and scale add predictive power to the decision to admit. This is particularly true in maintaining equitability between those referred from different locations.

## Background

We have shown that a structured professional judgment instrument for allocating patients to the appropriate level of therapeutic security has good psychometric properties and can distinguish those admitted from a remand prison to various levels of therapeutic security with good receiver operating characteristics, sensitivity and specificity in a retrospective-cohort study [1,2]. The same scale had predominantly static rather than dynamic characteristics and measured the clinical need for therapeutic security and specialist interventions that is distinct from measures of risk [3]. The assessment of need for therapeutic security can be understood as determining who would benefit from admission to a forensic mental health service or to various levels of therapeutic security. Once accepted onto a waiting list for admission to a given level of therapeutic security however, the prioritising of those on the waiting list may be determined by different considerations such as the urgency of need for treatment and legal imperatives. The criteria for deciding who may be moved to a less secure/intensive care place or discharged are also distinct from the admission triage criteria [3]. We set out to validate a structured professional judgment instrument for prioritising patients who had been accepted onto the waiting list for admission to a medium and high secure psychiatric in-patient unit.

Similar instruments for selecting and allocating to waiting lists have been devised for interventional cardiology [4-7], transplant surgery [8,9] and other areas of practice. Ethical guidance for how to structure such decision making processes emphasises four conditions for accountability of reasonableness [10] namely openness, relevance, an appeals mechanism and enforcement. The first two of these can be seen as arguments in favour of the development of structured professional judgment instruments such as this. Debate regarding the second two criteria [11] is beyond the scope of this work at this stage.

Based on our clinical experience, we hypothesised that when organising a waiting list, two decisions must be made. The first decision is to assess whether the patient referred for admission needs this level of service - in this case, admission to a high and medium secure hospital; the second decision concerns who amongst those who have qualified to go on the waiting list should be prioritised. This second decision is not always explicitly formulated and it is not clear that it is based on the same criteria as the first. Prioritisation, unlike assessment of need, is likely to change from week to week, and should be a dynamic rather than a static assessment, repeated as often as appropriate. The interaction between the relatively static assessment of need for therapeutic security and the dynamic assessment of the urgency of need is also unclear. It could be argued that those who meet criteria for admission to a given level of therapeutic security should be prioritised over those who do not meet the criteria. This is implicit when those who do not meet criteria are excluded from a waiting list. However the urgency of need when prioritising those who are accepted onto a waiting list may be determined by many other criteria. We set out to draft and validate a set of criteria for decision making regarding the prioritisation of the waiting list for the Central Mental Hospital, the only forensic mental health facility in the Republic of Ireland. We have followed the criteria suggested for validation of risk assessment instruments, though this is not itself a risk assessment instrument [12].

## Methods

### Study Design

This study consisted of three phases. The first was an iterative drafting process followed by an observational study of decision making in practice at the weekly referrals meeting when all referrals are discussed, accepted for admission or dealt with in some other way, and those accepted are prioritised. This has been described elsewhere [2]. The structured professional judgment instrument described here - the DUNDRUM-2 triage urgency instrument is part of the 22^nd ^revision of this draft. It forms part of a suite of structured professional judgment instruments [1] along with the DUNDRUM-1 triage security instrument for assessing the level of therapeutic security required [2], and two instruments for assessing readiness for movement to less secure places, the DUNDRUM-3 programme completion instrument and the DUNDRUM-4 recovery instrument [3].

The third phase was a prospective study in which all those on the waiting list over a six month period from January to June 2010 were rated using the DUNDRUM-1 and DUNDRUM-2 each week based on pre-admission written and oral assessment information presented at the weekly referrals meeting, supplemented where necessary by further information obtained from the clinicians presenting the referrals. The consultant psychiatrists chairing the meeting and the clinical director whose role was to resolve any impasses were blind to the ratings at the time when prioritisation decisions were made.

### Setting

The Central Mental Hospital provides high, medium and low therapeutic security and community follow-up services for a population of 4.4 million. At the time of the study there were 93 in-patient beds at varying levels of therapeutic security. The service also provides extensive mental health in-reach services to the busiest remand and sentenced committals prisons (prisons that accept new admissions to prison) in the state, and to the other prisons. Patients can be admitted to the hospital from the prisons under the Criminal Law (Insanity) Act 2006 if medically certified. They can also be admitted from the courts if found unfit to stand trial or not guilty by reason of insanity. We have previously described the process of diversion from prison custody [2]. In addition the service provides a tertiary referral and assessment service for local community mental health teams and admits patients under the civil Mental Health Act on transfer from local psychiatric hospitals. Clinicians present cases assessed for admission at a weekly referrals meeting where new referrals are allocated, as described above.

The DUNDRUM-2 triage urgency scale was rated by GF based on the pre-admission assessments presented at the weekly meeting. GF also rated the DUNDRUM-1 triage security items. Data were collected over a six month period from January to June 2010.

### Rating Scale: DUNDRUM-2 triage urgency scale

The DUNDRUM-2 triage urgency scale is intended to be used only for those who have been accepted onto the waiting list. The criteria for being accepted on the waiting list are covered by the DUNDRUM-1 triage security instrument [2,3]. The DUNDRUM-2 triage urgency instrument consists of six operationally defined items. These are (i) issues concerning the current location, (ii) mental health, (iii) suicide prevention, (iv) humanitarian and human rights considerations, (v) systemic issues and (vi) legal urgency [Additional file 1]. Because those accepted onto the waiting list may while on the waiting list be in the community (e.g. conditionally discharged forensic patients), in a remand prison, serving a sentence, requiring a move to a more secure place from a less secure hospital or require a move from a more secure or equally secure place in another hospital, the first item has a set of ratings for each of these five locations and the patient is rated only for the one that corresponds to their current location. The remaining five items are rated for all patients on the waiting list. Each item is rated 0 to 4 according to operationally defined criteria, where '4' indicates a need for immediate admission and '0' indicates that there is no current need for admission. The six items are then added to calculate a score for the purposes of these validation studies.

We have trained mental health professionals from psychiatry, nursing, psychology, social work and occupational therapy to use the DUNDRUM-1 and DUNDRUM-2 by guiding the use of the handbook followed by joint ratings of three prepared vignettes. This training takes two and a half hours and forms part of the induction for all new clinical staff, with optional top up training every six months.

### Outcome Measures

The key outcome measure is whether or not the person was admitted to the Central Mental Hospital. Not all those accepted onto the waiting list are admitted. Over time their clinical state may improve, they may be released by the courts on bail, come to the end of a sentence or they may be diverted via the courts to some other less secure hospital or community placement. Of the 16 remand prisoners initially placed on the waiting list but not eventually admitted, 14 were diverted to other less secure hospitals or community mental health teams via the courts. Accordingly the rating for the DUNDRUM-2 was compared for the time when the person was admitted or for the last week when they were removed from the waiting list. Comparisons were also made for when the person was first placed on the waiting list and when they were removed either because they were admitted to the Central Mental Hospital or were diverted or released or recovered.

### Statistics

All data was entered in SPSS-16. Inter-rater reliability for scale scores was calculated using exact probability calculations for agreement for each item. Internal consistency was examined using factor analysis and Cronbach's alpha statistic. Cross validation was examined using Spearman's rank correlation coefficient, a non-parametric test. The relationship between the DUNDRUM-1 score and admission as outcome was examined using univariate general linear modelling with admission as dependent variable, the DUNDRUM-2 score as fixed factor and location prior to admission as co-variant. One-way analysis of variance was then used to distinguish between sub-groups. The receiver operating characteristic (ROC) area under the curve (AUC) was calculated with 95% confidence intervals taking as the null hypothesis that AUC = 0.5. For item to outcome tests of criterion validity, those admitted were compared with those not admitted using univariate analysis of variance and X^2 ^tests. Change over time for individual items was examined with paired t-tests.

## Results

### Sample

During the six month observation period 65 individuals were placed on the waiting list and 38 were eventually admitted, some after the end of the six month observation period. There were 10 women placed on the waiting list of whom 6 were admitted and 56 men of whom 32 were admitted. Women were no more likely to be admitted than men (X^2 ^= 0.03, NS, Fisher's exact probability test = 1.0, NS).

### Inter-rater reliability

Twenty four patients were rated independently by a second clinician (CO'N). Mean scores were 12.4 (SD 2.9) and 12.5 (SD 3.7) for the two raters (paired t-test t = -0.21, df = 23, NS). The kappa statistic can only be calculated for items rated 0/1. Each item of the DUNDRUM-2 triage urgency scale is rated from '0' to '4' so that any two raters may agree randomly in 20% of cases. There was exact agreement in the rating for items 1 'location' in 11/24 (46%, binomial exact probability p < 0.004); item 2 'mental health' 10 (42%, p < 0.013), item 3 'suicide prevention' 9 (38%, p = 0.036), item 4 'humanitarian considerations' 12 (50%, p < 0.001), item 5 'systemic considerations' 24 (100%, p < 0.001) and item 6 'legal urgency' 12 (50%, binomial exact probability p < 0.001). The two raters differed by no more than one point (random probability 52%) in 18/24 (75%, p = 0.019) for item 1, 22 (92%, p < 0.001) for item 2, 19 (79% p = 0.006) for item 3, 18 (75% p = 0.019) for item 4, 21 (88% p < 0.001) for item 5 and 23 (96% p < 0.001) for item 6.

### Internal consistency

The first item of the DUNDRUM-2 (current location) was defined separately for each of five possible locations (community, remand prison or court, sentenced prison, less secure hospital, equal or more secure hospital) but was treated as a single item for this initial analysis. An initial factor analysis of the six items rated in the DUNDRUM-2 yielded two components with Eigenvalue greater than 1. The first component had an Eigenvalue = 3.2 accounting for 50% of the variance and the second component had Eigenvalue = 1.3 accounting for 21% of the variance. Principle components analysis showed that the first five items loaded positively on the first component (all r > 0.6) with only the sixth item 'legal urgency' loading negatively. The second component loaded positively on the first item 'location' and the sixth item 'legal urgency' with the remaining items loading negatively. Further inspection showed that 'legal urgency' correlated with the 'current location' rating for remand prisoners but not for other locations.

Cronbach's alpha is a measure of internal consistency. For all 65 patients on the waiting list, treating the first item 'current location' as a single item yielded alpha = 0.723. Each of the first five items if deleted lead to small decreases in the alpha statistic and the sixth item 'legal urgency' if deleted lead to an increase in Cronbach's alpha from 0.723 to 0.814. Cronbach's alpha calculated for the 39 persons in custody on remand was alpha = 0.754, while deletion of any item lead to negligible declines in the alpha statistic except for the sixth item 'legal urgency' which if deleted lead to an increase in alpha to 0.802. Calculating Cronbach's alpha for the 21 persons serving prison sentences yielded alpha = 0.681. As before deletion of any one item lead to very small declines in the alpha statistic except for the sixth item 'legal urgency' deletion of which improved the alpha statistic to 0.811. Similarly for the seven patients referred from less secure psychiatric hospitals, Cronbach's alpha = 0.821. Deleting items lead to very small falls in the alpha statistic except for the third item (suicide risk) deletion of which caused alpha to increase to 0.830, a very small increase, and the sixth item 'legal urgency' deletion of which increased alpha to 0.888.

### Cross-validation

For the 65 patients on the waiting list, the DUNDRUM-2 triage urgency score correlated positively with the DUNDRUM-1 triage security score (Spearman r = +0.663, p < 0.001). Factor analysis of the six items of the DUNDRUM-2 combined with the eleven items of the DUNDRUM-1 for all 65 patients generated five components with Eigenvalue greater than 1. The first had Eigenvalue = 6.9 accounting for 40.8% of the variance and all items loaded positively on it except triage urgency item 6 'legal urgency'. The second component had Eigenvalue = 2.6 accounting for 15.2% of the variance and loaded strongly positively on triage urgency item 3 'suicide prevention', triage security item 2 'seriousness of self harm' and triage security item 4 'immediacy of risk of suicide/self harm'. The third component had an Eigenvalue of 1.6 accounting for 9.4% of the variance and loaded negatively on Triage urgency items 2 'mental health' and 4 'humanitarian issues' and loaded positively on triage security items 2 'seriousness of deliberate self harm' and 8 'victim issues'. This component appears to lack any coherent theme. The fourth component had an Eigenvalue of 1.2 accounting for 7.3% of the variance and loaded positively on triage urgency item 6 'legal urgency' and triage security item 11 'legal procedure'. The fifth component with Eigenvalue 1.1 accounting for 6.3% of the variance had no coherent theme. Because the first component appears to include almost all items and the remaining components are thematically subsets such as 'suicide management' and 'legal considerations' it would appear that there is no factorial distinction between the DUNDRUM-1 and DUNDRUM-2 items. In keeping with this, testing the internal consistency of a combined scale of 17 items has Cronbach's alpha = 0.861. Omitting any one item lead to small decreases in the alpha statistic (lowest 0.849) except for triage urgency item 6 'legal urgency' omission of which lead to an increase of the alpha statistic to 0.876, a negligible increase.

### Location

Table 1 shows that of the 65 accepted onto the waiting list in the six month observation period, 38 were in prison on remand awaiting trial (22 of whom were admitted), 20 were serving a prison sentence (12 of whom were admitted) and 7 were in another less secure psychiatric hospital (4 of whom were eventually admitted). No location had a proportion admitted that was significantly different from any other location (X^2 ^= 0.1, df = 2, NS).

**Table 1:** DUNDRUM-2 triage urgency scores by location at time accepted onto waiting list

Location	Not admitted			Admitted			All		
	**mean**	**sd**	**n**	**mean**	**Sd**	**n**	**mean**	**Sd**	**N**

Remand	5.4	4.1	16	12.1	3.4	22	9.3	4.9	38

Sentenced	11.5	2.1	8	12.9	3.4	12	12.4	2.9	20

Less secure hospital	11.0	3.5	3	13.5	1.3	4	12.4	2.6	7

All	7.8	4.5	27	12.5	3.2	38	10.6	4.4	65

General linear model effect for admitted or not admitted F = 11.0, df = 1, p = 0.002; effect for location F = 8.2, df = 2, p = 0.001; interaction of admission and location F = 4.3, df = 2, p = 0.018.

In an overall estimate of effect sizes using general linear modelling with admission as the dependent outcome variable and location prior to admission as the independent variable, significant effects were found for admission (F = 11.0, df = 1, p = 0.002) and location (F = 8.2, df = 2, p = 0.001) and the interaction between admission outcome and location (F = 4.3, df = 2, p = 0.018). Table 1 shows that at the time when placed on the waiting list, there were significant differences in base line on the DUNDRUM-2 triage urgency score between all those on remand compared to all those sentenced or in less secure hospitals (ANOVA F = 4.4, df = 2, p = 0.016). Those admitted from the remand prisons had higher scores than those not admitted from remand prisons, while those admitted from prison who were serving sentences or who were admitted from other less secure hospitals had only marginally higher scores than those not admitted.

A different pattern emerged when the DUNDRUM-2 score was examined at the time when the person left the waiting list (a mean of 23.9 days (S.D. 35.9) after first being placed on the waiting list). People left the waiting list either because they were admitted to the Central Mental Hospital or were thought to no longer need admission to the hospital. Table 2 shows that although the three locations did not differ significantly overall by then (ANOVA F = 0.9, df = 2, NS), those not admitted had consistently lower scores than those admitted for each location, while there was no significant difference between any one location and the other locations (General linear model estimates of effect for admission F = 33.2, df = 1, p < 0.001, location F = 1.9, df = 2, NS and the interaction of admission and location was also not significant). Comparing those not admitted at baseline (Table 1) and when exiting the waiting list (Table 2) shows that the DUNDRUM-2 is a dynamic measure which is sensitive to change.

**Table 2:** DUNDRUM-2 triage urgency scores by location at time when removed from waiting list, whether by admission or not

Location	Not admitted			admitted			All		
	**mean**	**Sd**	**n**	**mean**	**sd**	**n**	**mean**	**Sd**	**N**

Remand	4.4	2.9	16	12.1	3.3	22	8.9	4.9	38

Sentenced	6.0	3.4	8	12.8	5.0	12	10.1	5.5	20

Less secure hospital	8.3	4.2	3	13.5	1.3	4	11.3	3.8	7

All	5.3	3.4	27	12.5	3.7	38	9.5	5.0	65

General linear model effect for admitted or not admitted F = 33.2, df = 1, p < 0.001; effect for location F = 1.9, df = 2, NS; interaction of admission and location F = 0.4, df = 2, NS.

The DUNDRUM-1 triage security items may also influence the decision to admit. Table 3 shows that at baseline when first placed on the waiting list, those referred from less secure hospitals had higher scores than remand or sentenced prisoners (ANOVA F = 4.6, df = 2, p = 0.014). Those not admitted from remand prisons had lower scores than those who were eventually admitted from remand prisons but there was no difference between those admitted and not admitted for sentenced prisoners and those in less secure hospitals. The general linear model estimate of effect size was however significant overall for admission outcome (F = 4.4, df = 1, p = 0.04), larger

**Table 3:** DUNDRUM-1 triage security scores by location at time placed on waiting list

Location	Not admitted			admitted			All		
	**mean**	**Sd**	**n**	**mean**	**sd**	**n**	**mean**	**Sd**	**N**

Remand	15.2	6.1	16	25.9	6.3	22	21.4	8.1	38

Sentenced	21.4	5.3	8	22.8	6.8	12	22.2	6.1	20

Less secure hospital	30.3	2.5	3	30.3	2.9	4	30.3	2.5	7

All	18.7	7.4	27	25.3	6.5	38	22.6	7.6	65

General linear model effect for admitted or not admitted F = 4.4, df = 1, p = 0.04; effect for location F = 7.7, df = 2, p = 0.001; interaction of admission and location F = 5.0, df = 2, p = 0.01.

for location (F = 7.7, df = 2, p = 0.001) and the interaction between admission outcome and location was significant (F = 5.0, df = 2, p = 0.01).

When the DUNDRUM-1 triage security scores are compared at the time of leaving the waiting list however (Table 4), significant differences emerge between those admitted and not admitted for both remanded and sentenced prisoners. Those admitted from remand and sentenced prisons had higher scores than those not admitted. Those referred from less secure hospitals still had higher scores than those from the other two locations, with no difference between those admitted from less secure hospitals and not admitted (general linear model estimate of effect size for admission outcome F = 7.6, df = 1, p = 0.008, effect for location F = 7.7, df = 2, p < 0.001 and the interaction between admission outcome and location F = 3.5, df = 2, p = 0.037).

**Table 4:** DUNDRUM-1 triage security scores by location at time when removed from waiting list, whether by admission or not

Location	Not admitted			admitted			All		
	**mean**	**Sd**	**n**	**mean**	**sd**	**n**	**mean**	**Sd**	**N**

Remand	13.9	6.9	16	25.9	6.3	22	20.8	8.8	38

Sentenced	17.5	7.4	8	22.6	7.0	12	20.6	7.4	20

Less secure hospital	30.3	2.5	3	30.3	2.9	4	30.3	2.5	7

All	16.8	8.3	27	25.3	6.6	38	21.8	8.4	65

General linear model effect for admitted or not admitted F = 7.6, df = 1, p = 0.008; effect for location F = 7.7, df = 2, p < 0.001; interaction of admission and location F = 3.5, df = 2, p = 0.037.

### Change over time

The mean time spent on the waiting list before being either admitted or removed for some other reason was 23.9 days (S.D. 35.9). Those admitted spent a mean of 21.4 days on the waiting list (SD 40.7) while those not admitted spent a mean of 27.3 days on the waiting list (SD 29.2), a difference that was not statistically significant.

The DUNDRUM-2 triage urgency score changed while on the waiting list, from a mean score of 10.6 (S.D. 4.4) when first placed on the waiting list to 9.5 (S.D. 5.0) by the time the person was removed from the waiting list, either because they were admitted, diverted or no longer needed admission (mean difference 1.1 (S.D. 3.1, paired t = 2.7, df = 64, p = 0.007). The DUNDRUM-1 triage security score changed relatively less over the same period from a mean of 22.6 (S.D. 7.6) to 21.8 (S.D. 8.4) which did not reach statistical significance (mean difference 0.8 (S.D. 3.4) paired t = 1.9, df = 64, NS).

To clarify the differences over time, Table 5 shows that for the DUNDRUM-1 triage security scale, those not admitted had statistically significant falls in scores between the time they were first placed on the waiting list and the time they exited the waiting list, while those admitted did not have any decline in their scores. Sentenced prisoners had the largest declines in their scores. Table 6 shows that for the DUNDRUM-2 triage urgency score, remand and sentenced prisoners who were not admitted had falls in their scores but those who were admitted did not have such falls.

**Table 5:** DUNDRUM-1 triage security scores by location, change between time placed on waiting list and time when removed from waiting list, whether by admission or not

Location	Not admitted			admitted			All		
	**Mean change**	**sd**	**n**	**Mean change**	**sd**	**n**	**Mean change**	**sd**	**N**

Remand	0.9	3.4	16	0.0	0.2	22	0.4	2.2	38

Sentenced	5.5	2.8	8	0.1	3.6	12	2.3	4.2	20

Less secure hospital	2.7	3.1	3	0.0	0.0	4	1.1	2.3	7

All	2.5	3.7	27	0.1	1.9	38	1.1	3.1	65

General linear model effect for admitted or not admitted F = 13.4, df = 1, p < 0.001; effect for location F = 4.9, df = 2, p = 0.01; interaction of admission and location F = 4.8, df = 2, p = 0.011.

**Table 6:** DUNDRUM-2 triage urgency scores by location change between time placed on waiting list and time when removed from waiting list, whether by admission or not

Location	Not admitted			admitted			All		
	**Mean change**	**sd**	**n**	**Mean change**	**sd**	**n**	**Mean change**	**sd**	**N**

Remand	1.3	4.5	16	0.0	0.2	22	0.5	2.9	38

Sentenced	3.9	6.7	8	0.2	0.9	12	1.7	4.5	20

Less secure hospital	0.0	0.0	3	0.0	0.0	4	0.0	0.0	7

All	1.9	5.1	27	0.0	0.5	38	0.8	3.4	65

General linear model effect for admitted or not admitted F = 2.7, df = 1, p = 0.044; effect for location F = 1.5, df = 2, p < 0.048; interaction of admission and location F = 1.2, df = 2, p = 0.038.

### Outcome - triage for admission and receiver operating characteristics

The receiver operating characteristic (ROC) was calculated for the DUNDRUM-2 triage urgency score where the outcome is admission, and also for the DUNDRUM-1 triage security score and the combined score which is the sum of the two. This was done for the ratings at the time when patients were placed on the waiting list, and also at the time when patients were removed from the waiting list, either because they had been admitted or no longer needed admission to the Central Mental Hospital. Table 7 shows that at baseline, all three had an area under the curve (AUC) that was significantly better than chance. The combined score was marginally better than the other two, though the confidence intervals for all three overlapped. At the time of admission or removal from the waiting list however, the triage urgency scale had the highest AUC (0.912, 95% CI 0.838 to 0.986), while the other two also had AUCs significantly better than chance and all three were greater than at baseline.

**Table 7:** Receiver operating characteristics

	Area under the curve (AUC)	**S.E**.	95% confidence interval	
Scores by location at time accepted onto waiting list				

DUNDRUM-1 triage urgency score	0.788	0.058	0.674	0.901

DUNDRUM-2 triage security score	0.755	0.063	0.632	0.879

Combined D-1 and D-2	0.792	0.057	0.679	0.904

scores by location at time when removed from waiting list, whether by admission or not				

DUNDRUM-1 triage urgency score	0.912	0.038	0.838	0.986

DUNDRUM-2 triage security score	0.789	0.059	0.673	0.905

Combined D-1 and D-2	0.869	0.044	0.782	0.955

All areas under the curve (AUC) significantly greater than 0.5 at p < 0.001 where the null hypothesis is that the area under the curve is 0.5.

Because the first item of the DUNDRUM-1 triage urgency scale is defined separately according to the location of the patient while on the waiting list, the receiver operating characteristic was calculated separately according to the location of the patient for the ratings at the time the patient left the waiting list. Table 8 shows that for those on remand, the DUNDRUM-2 triage urgency score, DUNDRUM-1 triage security score and the combined score all had AUC significantly greater than 0.5 (p < 0.001 for all three). For those serving prison sentences, the DUNDRUM-1 triage urgency score and the combined score had AUC significantly greater than 0.5 (p < 0.01 for each), Although the DUNDRUM-2 triage security score had AUC = 0.724, the 95% confidence interval overlapped 0.5. For the 7 patients in less secure hospitals, only the combined score was significantly greater than 0.5 (AUC 0.958, p = 0.052). This probably reflects the small sample size.

**Table 8:** Receiver operating characteristics

	Area under the curve (AUC)	**S.E**.	95% confidence interval	
Remand prisoners, n = 38, 22 admitted, 16 not admitted.				

DUNDRUM-1 triage urgency score	0.942	0.038	0.868	1.000

DUNDRUM-2 triage security score	0.905	0.050	0.806	1.000

Combined D-1 and D-2	0.940	0.038	0.864	1.000

Sentenced prisoners, n = 20, 12 admitted, 8 not admitted				

DUNDRUM-1 triage urgency score	0.885	0.082	0.725	1.000

DUNDRUM-2 triage security score	0.734	0.118	0.493	0.954

Combined D-1 and D-2	0.844	0.093	0.661	1.000

Patients in less secure hospitals, n = 7, 4 admitted, 3 not admitted				

DUNDRUM-1 triage urgency score	0.875	0.145	0.591	1.000

DUNDRUM-2 triage security score	0.500	0.234	0.042	0.958

Combined D-1 and D-2	0.958	0.077	0.808	1.000

All areas under the curve (AUC) significantly greater than 0.5 at p < 0.001 where the null hypothesis is that the area under the curve is 0.5.

### Outcome - triage for admission and individual items

Table 9 shows that for ratings at the time of admission or removal from the waiting list, each item of the DUNDRUM-2 triage urgency items had higher mean scores for those admitted using analysis of variance. Table 9 also shows that higher ratings were associated with admission using the Chi-squared statistic. Two items however were of marginal statistical significance, item TU3 'self harm' and item TU6 'legal urgency' (both 0.05< p < 0.1). For the eleven items of the DUNDRUM-1 triage security scale, only items TS2 'seriousness of self harm' and item TS3 'immediacy of self harm' were marginally or not significantly associated with admission. Item TS11 'legal procedure' had by far the strongest statistical association with eventual admission.

**Table 9:** Item to outcome analysis for DUNDRUM-1 and DUNDRUM-2 scales

	Not admitted		Admitted		ANOVA, df = 1	X^2^ df = 4
**Item**	**mean**	**S.D**.	**mean**	**S.D**.	**F**	**X^2^**

DUNDRUM-2 triage urgency items						

TU1 location	1.1	0.8	2.7	0.9	49.3^4^	41.3^4^

TU2 mental health	0.9	1.1	1.3	1.2	23.1^4^	20.0^4^

TU3 self harm	0.9	1.1	1.3	1.2	2.9^1^	7.9^1^

TU4 humanitarian	0.1	0.4	1.8	1.6	28.6^4^	20.9^4^

TU5 systemic	1.6	1.7	3.5	1.1	31.3^4^	25.6^4^

TU6 legal urgency	0.6	0.5	1.0	1.1	3.6^1^	6.7

DUNDRUM-1 triage security items						

TS1 serious violence	1.5	1.1	2.5	1.1	13.2^4^	14.7^3^

TS2 serious self harm	1.2	1.1	1.2	1.2	0	7.8^1^

TS3 immediacy of violence	1.6	1.0	2.7	1.2	14.9^4^	13.6^3^

TS4 immediacy of self harm	0.9	1.2	1.2	1.4	0.7	7.4

TS5 special forensic need	1.2	0.9	2.2	1.0	15.9^4^	13.5^3^

TS6 absconding	1.6	0.9	2.3	1.0	6.8^2^	10.1^2^

TS7 preventing access	1.9	0.9	2.5	0.8	7.3^3^	7.3

TS8 victim issues	1.7	1.2	2.5	1.0	7.8^3^	14.0^3^

TS9 risk of violence	1.8	1.1	2.6	0.9	12.2^4^	11.5^2^

TS10 institutional behaviour	1.4	0.8	1.9	1.1	3.9^2^	9.1^1^

TS11 legal procedure	1.9	1.5	3.7	0.5	43.9^4^	32.4^4^

.^1 ^p < 0.1; ^2 ^p < 0.05; ^3 ^p < 0.01; ^4 ^p < 0.001.

Concerning change over time, paired t tests showed significant falls in mean scores for DUNDRUM-2 item 1 'location' (paired t = 2.1, df = 64, p = 0.006), item 2 'mental health' (paired t = 2.1, p = 0.04), item 4 'humanitarian issues' (paired t = 1.8, p = 0.08) and item 5 'systemic' (paired t = 2.5, p = 0.015). Item 3 'self harm' and item 6 'legal urgency' had declines which did not reach significance. For the DUNDRUM-1 triage security items there were significant declines only for item 3 'immediacy of violence' (paired t = 2.2, df = 64, p = 0.031) and item 11 'legal procedure' (paired t = 1.8, p = 0.031).

## Discussion

The DUNDRUM-2 triage urgency scale has good psychometric properties. It has good inter-rater reliability and high internal consistency although the legal urgency item is the least cohesive of the six items. The DUNDRUM-2 triage urgency score correlated with the DUNDRUM-1 triage security score. It appears for practical purposes that there is only a single underlying 'factor' amongst all six items of the DUNDRUM-2 triage urgency scale and the eleven items of the DUNDRUM-1 triage security scale. However both the content and the 'dynamic' change sensitive nature of the DUNDRUM-2 triage urgency items provide sufficient reason for distinguishing the DUNDRUM-1 from the DUNDRUM-2 scale. This is because the DUNDRUM-1 has distinct content oriented towards assessing need rather than urgency. It is interesting to note that although in this study there were some falls over time in the DUNDRUM-1 score, this arose only due to changes in the items regarding immediacy of risk of violence and legal urgency.

When predicting admission from the waiting list the DUNDRUM-2 triage urgency score generally has a better area under the curve of the receiver operating characteristic compared to the DUNDRUM-1 triage security score and the combined score (Tables 7 and 8). This is a property of the instrument rather than a property of the population, and suggests that the instrument would work in a different population. Because of the legal and other structural differences between jurisdictions however, validation in each jurisdiction where it is proposed to use the instruments would be necessary to establish the receiver operating characteristics.

Each item of the DUNDRUM-1 triage security scale and the DUNDRUM-2 triage urgency scale was tested for its relationship to outcome (admitted or not admitted from the waiting list). Table 9 shows that all items of the DUNDRUM-2 triage urgency scale were greater in those who were admitted, while all items of the DUNDRUM-1 triage security scale except item 4 'immediacy of self harm' were also greater in those who were admitted.

### Systems issues

In the course of this study it emerged that there is an anomaly concerning patients referred from hospitals at lower levels of security. Those accepted onto the waiting list from less secure hospitals had higher scores on the DUNDRUM-1 triage security scale than those accepted from other locations (Table 3) and those from less secure hospitals who were admitted had the same scores on the DUNDRUM-1 as those from less secure hospitals who were not admitted (Table 4). Others have shown that where forensic therapeutically secure beds are scarce, those admitted from less secure hospitals are disadvantaged [13]. However the DUNDRUM-2 triage urgency scale demonstrated equity between patients referred from different locations - those admitted from different locations had the same mean scores on the DUNDRUM-2 scale (Tables 1 and 2) and at the time of admission those admitted from less secure hospitals had higher scores on the DUNDRUM-2 than those not admitted from less secure hospitals (Table 2).

There is a clinical justification for having a lower admission threshold for those in prison compared to those already in another hospital. Those in another hospital are in receipt of mental health care, they have the protections of treatment under mental health legislation where necessary and they are not in the challenging and anti-therapeutic environment of a prison. These are all 'urgency' factors rather than factors concerning need for a particular level of therapeutic security. However it may be that there is reluctance to admit patients from less secure or other hospitals for another reason. Such patients commonly have much longer lengths of stay in conditions of high or medium therapeutic security. Clinicians and bed managers may be reluctant to admit those who are likely to become long-stay as they will reduce the capacity of the service to admit future incident cases including those who are in urgent need in prisons. We have not included an item to take account of likely length of stay as a 'systemic' consideration because we have insufficient evidence on which to base an operationalised rating scale that would accurately predict likely length of stay. Likely length of stay was included in earlier structured professional judgment instruments for assessing need for therapeutic security [14,15] though these lacked definitions. Some preliminary descriptions of those needing longer periods in medium security include treatment resistance, co-morbidity, chronic challenging behaviour, need for support and dependency [16-18].

### Decision making structures and structured professional judgment

Another reason for inconsistency in waiting list prioritisation which can be discounted in this study has been described [19] in complex systems where patients from different sources compete for intensive care places. In the system described by Cooper et al [19] problems arose when multiple decision makers had access to the same beds by parallel tracks functioning independently of each other, when communication between decision makers was via indirect routes and when guidelines were unclear or not consistently applied. Cooper et al [19] concluded that where there is a high census or full occupancy, decision makers must prioritise admissions using the 'triage' mode. Full occupancy describes the operating conditions for the waiting list in this study and in forensic secure services generally. To replicate this study, it would be essential to note that although all those admitted had a preadmission assessment by senior nurses as well as doctors, all those placed on the waiting list had been assessed by a consultant forensic psychiatrist and the decision to admit was taken by the duty consultant forensic psychiatrist at a weekly referrals meeting attended by all clinicians involved, with impasses and urgent decisions referred to the clinical director. The decision making process was therefore clear, as recommended by Daniels and Sabin [10] and this is likely to have improved consistency.

The DUNDRUM-2 triage urgency items have been validated as a scale but it has been developed for use as a structured professional judgment instrument, not an actuarial scale. Structured professional judgment instruments are intended to guide and assist the decision makers, but they are not intended to bind the decision maker in the way an actuarial checklist score might appear to do. The advantage of using a structured professional judgment instrument over unstructured professional judgment should be the transparency of the decision making process and openness to audit at the systems level and review at the individual level, in keeping with recommendations for ethical rationing [10,11,19]. Rid [11] has criticised Daniels and Sabin's [10] approach on the grounds that it is insufficiently legalistic. We are sceptical of this as a valid criticism on pragmatic grounds. Applying structured professional judgment instruments within a transparent decision making structure as described here should remedy a number of problems described by Cooper et al [19] due to multiple decision makers, parallel tracks for admission that function independently of each other, indirect communication between multiple decision makers and lack of guidelines or failure to enforce guidelines. Cooper et al [19] suggest that remedying such problems would lead to better equity, accountability and efficiency of resource allocation, with improved capacity as a result. Use of a reliable and validated structured professional judgment instrument should lead to an improvement in the consistency and reliability of decision making between clinicians and between centres. Mechanisms for decisions, reviews and appeals will differ from jurisdiction to jurisdiction. This instrument should enable those conducting such panels or hearings to make decisions based on the clarity and transparency of the structured professional judgement approach particularly when weighing the evidence of different experts or resolving differences between experts.

### Limitations

The numbers included in this prospective naturalistic outcome study are small when some sub-groups are considered, particularly for those waiting for admission from less secure hospitals. All other analyses had sufficient power to reach statistical significance and there does not appear to be any evidence of possible error due to lack of statistical power. Although the receiver operating characteristic is a property of the instrument not the population, this structured professional judgement instrument will have to be tested and validated in other jurisdictions where the organisation and structure of forensic mental health services may differ as may legal frameworks and processes. We believe however that the item content is likely to be generalisable.

## Conclusions

This naturalistic prospective observational study provides evidence that there is a distinction between the items assessing need for admission to various levels of therapeutic security such as the medium and high secure forensic hospital studied here and the items assessed to decide the prioritisation of those on a waiting list for admission to a medium or high secure forensic hospital.

## Competing interests

The authors declare that they have no competing interests.

## Authors' contributions

GF collected and entered data and coordinated the project. GF and CO'N rated the data and contributed to the item definitions in the manual. HGK produced the first draft of the manual, designed the study and carried out the data analysis. All contributed to the authorship of the paper and approved the final manuscript.

## Supplementary Material

Additional file 1 **DUNDRUM-2 triage urgency scale**. This is an extract from the handbook of the DUNDRUM Quartet. Click here for file
