# Supplementary material for: DUNDRUM-2: Prospective validation of a structured professional judgment instrument assessing priority for admission from the waiting list for a forensic mental health hospital
Source: BMC Res Notes. 2011 Jul 3;4:230. doi: 10.1186/1756-0500-4-230 (PMC3224499; doi:10.1186/1756-0500-4-230)
Supplement: Additional file 1 — DUNDRUM-2 triage urgency scale. This is an extract from the handbook of the DUNDRUM Quartet. [file 1756-0500-4-230-S1.PDF]

Additional files

Additional file 1 –.

DUNDRUM-2: Triage Urgency Items

These items are intended to provide a structured professional judgement instrument for prioritising those admitted from the waiting list to a therapeutically secure service. Those placed on the waiting list should be determined by the DUNDRUM-1 security triage items, though even this is a matter for clinical judgement and flexibility in the light of the patient's best interests.

The allocation of places is not a simple matter of first-come, first-served, assigning to each a place on the waiting list determined by the date the individual is first accepted onto the waiting list. In practice, when demand outstrips supply, other considerations prevail. Since demand for secure forensic in-patient places always outstrips supply, a chronological waiting list is never applied in practice. If the need for therapeutic security is more or less equal amongst those on a waiting list, then other considerations will determine urgency. Generally clinicians will prioritise those in prison over those who are already in a hospital elsewhere, and generally clinicians will prioritise those with the most life threatening current clinical needs over those who can safely be delayed on the waiting list or treated without admission. Further factors influencing the prioritisation of admissions include legal obligations and various pragmatic and systemic considerations concerning catchment areas and pathways through care. Systemic considerations may include contracting arrangements between public sector commissioners or insurance based funders of services and the state or independent sector providers of such services.

Lawyers may have difficulty with the concept of a non-chronological waiting list since they are accustomed to a prison system in which prison governors will invariably accept all those committed to custody by the courts, regardless of prison capacity and irrespective of the consequences for safety, over-crowding, and consequent adverse effects on the humane and therapeutic aspects of the milieu. It would not be possible to provide a hospital service on this basis, so that the purpose of committing to a hospital would be defeated if courts were given control over waiting lists or free access to hospitals irrespective of capacity or clinical need. Further, hospitals are accustomed to managing waiting lists, whether for elective treatment or emergency treatment, employing clinical triage decision making based originally on battlefield practice in which those most in need are prioritised over those who can wait or are less needy. On this basis, the ordering of the waiting list in forensic mental health practice prioritises those with mental disorders that cannot be effectively treated or managed in prison over those with minor illnesses or simple adjustment reactions to imprisonment itself. Those who need a given level of therapeutic security are prioritised over those who need a lesser level of therapeutic security (as in court diversion schemes).

The items which follow are commonly used as a means of prioritising cases for admission, other things being equal. It is assumed that the level of the level of therapeutic security required has already been assessed, as indicated by the DUNDRUM-1 Triage Security Items. Six items should be rated for each person on the waiting list. Because those considered for admission may be in the community, in remand or sentenced prisons or in other hospitals at higher or lower levels of therapeutic security, and because different considerations apply according to the current location, five alternative rating scales have been provided and labelled TU1A to TU1E, to indicate that only one rating should be counted for each patient. Cooper et al (2005) noted that having parallel tracks for admission was a major source of dysfunction in their admissions system. This item is intended to address this difficulty.

Priority due to mental health considerations (TU2), suicide prevention (TU3) and humanitarian considerations (TU4) are each given a domain for consideration, while systemic (TU5) and legal considerations (TU6) complete the scale of items.

Some prison governors may be deterred from reforming their regimes if poor practice (e.g. prolonged seclusion, failure to provide effective protection for vulnerable prisoners) is rewarded by the transfer of challenging or vulnerable prisoners to hospital. However there will be situations where a defendant or a prisoner with an undoubted mental disorder cannot be safely managed in a prison environment.

Note that in general, those in a lower level of therapeutic security are able to benefit from medical and nursing care in a therapeutic environment, whereas those in a prison are in a non-therapeutic environment that may be toxic to their mental health. Those in prison environments therefore usually take precedence over those in hospital environments.

TU1A: TRIAGE URGENCY: COMMUNITY FORENSIC PATIENT

This item is intended to give appropriate priority to those patients discharged from a forensic mental health service while required to comply with conditions and subject to recall. Wherever possible it is best to preserve the working alliance and to cultivate continuity of therapeutic relationships. Much may be agreed as part of the integrated care and treatment plan, with the advance preferences of the patient playing a significant part in how intervention is staged in the event of relapse or breach of conditions. The patient may prefer to be admitted to a local catchment area service or the patient may prefer to be readmitted to the forensic service, possibly to a pre-discharge ward rather than an admission ward if appropriate. However risk management must take precedence over patient preference where there is a clear divergence between the two.

TU1A: COMMUNITY FORENSIC PATIENT

0	No pre-admission assessment.
1	Essential elements of the community after-care and risk management package have broken down though the patient is not yet relapsing, exhibiting signature signs of risk scenarios or in breach of conditions of discharge.
2	Is relapsing or exhibiting signature signs of risk scenarios, or in breach of conditions of conditional discharge but current dynamic risk is sufficiently low to permit treatment in a lower level of therapeutic security, if necessary using the civil mental health act.
3	Is relapsing or exhibiting signature signs of risk scenarios, may not have breached conditions of discharge but working alliance and risk management are better served by a readmission to the forensic service
4	Is in breach of conditions of discharge, meets TS criteria for admission to this level of therapeutic security and dynamic risk factors are currently high .

Information Quality: □ no information; □ staff observation; □ interview; □ family informants; □ clinical records □ police records □ court papers □ other (tick all informants that apply)

TU1B: TRIAGE URGENCY: COURT/REMAND PRISONER

Remand prisoners have the highest psychiatric morbidity. Remand prisoners with severe mental illness may have been remanded for very minor or even nominal offences. Prison in-reach and court liaison / court diversion services exist to ensure that such persons are transferred to the appropriate community mental health facility or low secure unit at the earliest opportunity. Such patients are given a low priority for transfer to higher levels of therapeutic security, while not ruling out such a placement.

For those who have a triage security assessment indicating the need for a more secure placement, this item gives a higher weighting because placement in alternative community or lower secure places would not be appropriate. It is sometimes appropriate to consider those who have been refused bail because they are charged with a serious offence and at risk of a long sentence as if they were sentenced – see TU1C. This might lead to a rating of ‘3’ on that item rather than ‘2’ on this, and the person should be rated accordingly.

Those likely to be found unfit to stand trial (unfit to plead) should be accorded a high priority for transfer from prison to hospital so that they can be treated prior to trial. to be found unfit may result in a prolonged detention in forensic settings out of proportion to security need or risk assessment.

Those likely to be found not guilty by reason of insanity or made subject to a restriction order should similarly be given a high priority for admission, though not quite so urgently as those likely to be found unfit to stand trial. It is important that they should be treated and fully assessed prior to trial.

NB If there is no current mental disorder (broadly defined), the correct rating is zero (0), because the person is not in need of psychiatric admission or follow up.

TU1B: TRIAGE URGENCY: COURT/REMAND PRISONER

0	No pre-admission assessment **or** no current evidence of mental disorder.
1	Severe mental illness / mental disorder but can be diverted directly from court or from remand prison via court to a lower level of therapeutic security.
2	Severe mental illness / mental disorder and cannot be diverted directly to a lower level of therapeutic security (includes those who are refused bail / facing a long sentence if convicted)
3	Severe mental illness / mental disorder and may be found NGRI or made subject to a restriction order.
4	Severe mental illness / mental disorder and may be found unfit to stand trial

Information Quality: □ no information; □ staff observation; □ interview; □ family informants; □ clinical records □ police records □ court papers □ other (tick all informants that apply)

TU1C: TRIAGE URGENCY: SENTENCED PRISONER

Sentenced prisoners present different issues to remand prisoners. Legally, admission to a forensic secure placement is likely to be a requirement. There are often large numbers of mentally ill persons in prison who are serving sentences and are managed by prison in-reach mental health services in much the same way they would be managed in the community. The same rights to autonomy, beneficence and confidentiality apply in prison as in the community. Those who refuse treatment must have their wishes respected, unless they lack capacity and come within the definition of mental disorder in the appropriate mental health legislation, when the legal process under the relevant mental health legislation must be followed.

It is generally not appropriate to treat without consent in a prison. Transfer to hospital may in itself be enough to alleviate a mental disorder caused by the stress of imprisonment, and the ethical principle of reciprocity holds that when depriving an individual of all or part of their autonomy or freedom due to a mental disorder, there is an obligation to supply the means of alleviating the mental disorder to restore autonomy, preventing deterioration or optimising the quality of life while subject to any form of restriction due to mental disorder. This cannot be done in prison.

Particular priority should be accorded towards the end of a sentence if a community treatment and risk management package cannot be put in place by the prison in-reach mental health team.

NB If there is no current mental disorder (broadly defined), the correct rating is zero (0), because the person is not in need of psychiatric admission or follow up.

TU1C: TRIAGE URGENCY: SENTENCED PRISONER

0	No pre-admission assessment **or** no current evidence of mental disorder.
1	Can be treated and maintained by prison in-reach mental health team and after care plan can be put in place without transfer from prison to hospital.
2	Relapse of mental disorder in prison despite previous assessment, treatment, through care and aftercare plan delivered by in-reach mental health team.
3	Newly ascertained mental disorder, requires assessment, treatment, through care plan and community aftercare plan that cannot be completed in prison.
4	Near end of sentence, untreated and / or with no after-care or risk management plan in place in the community and this cannot be arranged in prison.

Information Quality: □ no information; □ staff observation; □ interview; □ family informants; □ clinical records □ police records □ court papers □ other (tick all informants that apply)

TU1D: TRIAGE URGENCY: PRIORITISING MOVES TO HIGHER LEVEL OF THERRAPEUTIC SECURITY

Transfers from a lower level of therapeutic security to a higher level may be required due to changed needs for therapeutic security per se (as assessed by the DUNDRUM-1 Triage Security items), due to an increase in assessed risk (e.g. as assessed by the HCR-20 dynamic items) or due to a specific need for specialist treatments. The ethical principles of proportionality and necessity should guide decision making. It should seldom if ever be necessary to move a patient up a level of therapeutic security only because of self harm or the prevention of suicide.

TU1D: TRIAGE URGENCY: PRIORITISING MOVES UP

NB If there is no current mental disorder (broadly defined), the correct rating is zero (0), because the person is not in need of psychiatric admission or follow up.

0	No pre-admission assessment **OR** no current evidence of mental disorder **OR** is not in another hospital
1	.Would benefit from a move to a higher level of therapeutic security in order to engage with specialist treatment programmes
2	Is in another hospital and meets TS criteria for a move to a level of therapeutic security intermediate between current location and a this (higher) level, but no intermediate placement is available (see also TU4).
3	Is in another hospital and meets TS criteria for a move to this (higher) level of therapeutic security (e.g. due to absconding or other TS items)
4	Is in another hospital and has exceeded the capacity of that hospital to safely care for the patient (e.g. may be subject to extraordinary measures see TU3) & meets TS criteria for a move to this (higher) level of therapeutic security.

Information Quality: □ no information; □ staff observation; □ interview; □ family informants; □ clinical records □ police records □ court papers □ other (tick all informants that apply).

TU1E: TRIAGE URGENCY: PRIORITISING MOVES TO SAME OR LOWER LEVEL OF THERAPEUTIC SECURITY

Patients have a right to be detained in no greater a degree of therapeutic security than is necessary and proportionate to their need. The DUNDRUM-1 TS items are a guide to this need. Risk assessment e.g. with the HCR-20 is complimentary to and adds to such an assessment. The DUNDRUM-3 Recovery and DUNDRUM-4 Programme Completion items are also a useful guide to readiness for moves to lower levels of therapeutic security. Moves from acute low secure (PICU) to longer term low secure units, or from medium term to longer term medium secure units may also be appropriate when progress in treatment is unlikely to lead to a move to a lower level of therapeutic security and in addition the quality of life is enhanced by such a move.

NB If there is no current mental disorder (broadly defined), the correct rating is zero (0), because the person is not in need of psychiatric admission or follow up.

TU1E: TRIAGE URGENCY: PRIORITISING MOVES TO SAME OR LOWER LEVEL OF THERAPEUTIC SECURITY

0	No pre-admission assessment **OR** no current evidence of mental disorder **OR** is not in another hospital.
1	Is in an out of catchment area hospital at the same level of therapeutic security and would benefit from a move to a hospital in the catchment area nearer to family and own community.
2	Is in another hospital (which may be out of catchment area) at the same or higher level of therapeutic security and would benefit from a move to this hospital (at the same or lower level) to engage in rehabilitation or family therapy programmes not available at the current placement, **OR** to have a better quality of life for longer term care at the same level of therapeutic security
3	Is in another hospital (which may be out of catchment area) at the same or higher level of therapeutic security and requires admission to this unit (at the same level of therapeutic security or a lower level) to connect with a pathway through care locally.
4	Is in another hospital at a higher level of therapeutic security and would benefit from a move to a lower level of therapeutic security (at this hospital) **OR** would benefit from a move to this specialised service at the same level of therapeutic security e.g. forensic intellectual disability service, acquired brain injury service.

Information Quality: □ no information; □ staff observation; □ interview; □ family informants; □ clinical records □ police records □ court papers □ other (tick all informants that apply).

TU2: TRIAGE URGENCY: MENTAL HEALTH

This item gives weight to clinical urgency, with life threatening problems taking precedence. Physical illness alone will not require admission to a therapeutically secure mental health unit and is better dealt with in a general hospital. In a general hospital security staff from the prison may be allocated to stay with the person or if the patient is already in a mental health service, staff from the mental health unit may be present by the bedside.

NB If there is no current mental disorder (broadly defined), the correct rating is zero (0), because the person is not in need of psychiatric admission or follow up.

TU2: TRIAGE URGENCY: MENTAL HEALTH

0	No pre-admission assessment **or** no current evidence of mental disorder.
1	Accepting treatment for severe mental illness in present place, whether in community, hospital or prison but would respond better or would benefit to a greater degree if transferred to hospital at this level of therapeutic security.
2	Stable but unsatisfactory mental health and cannot be treated for severe mental illness in present placement in a lower level of therapeutic security or in prison - e.g. in prison requires transfer under Mental Health legislation for treatment without consent.
3	Deteriorating mental state (psychosis) **and** physical state<br>**Either** in prison;<br>**OR** in current hospital placement due to lack of therapeutic security
4	A life-threatening state e.g. catatonic stupor or acute excited state that cannot be managed in prison or in current hospital at lesser level of therapeutic security

Information Quality: □ no information; □ staff observation; □ interview; □ family informants; □ clinical records □ police records □ court papers □ other (tick all informants that apply).

TU3: TRIAGE URGENCY: SUICIDE PREVENTION

This item is intended to give appropriate weight to those who need admission to hospital in order to manage the risk of suicide. An assessment that took account of risk (probability) only would prioritise many who engage in repetitive self harming behaviour that is of low lethality. The S-RAMM (Bouch & Marshall 2003, Ijaz et al 2009, Fagan et al 2009) identifies those who have preferred methods which are of high lethality. This item relies on the dichotomy between probability or immediacy on the one hand, and gravity (lethality) on the other. This emphasises the sensitivity of the item to change over time.

Traditionally, those remanded in custody charged with murder or rape were regarded as at high risk of completed suicide, particularly where the scenario is of a failed extended suicide. Failed 'suicide by cop' may also be a high risk. Brophy (2003) has shown that those charged with sex offences are at high risk of suicide, particularly those charged with offences against children. The same paper indicated however that the risk was higher for those still in the community, with those remanded in custody at no higher risk than other prisoners.

In general those who are already in hospital at any level of therapeutic security can be cared for sufficiently to prevent suicide e.g. by close nursing observations and detention under civil mental health legislation, though occasionally a high absconding risk may require admission to a low secure unit. Accordingly those already in a hospital are 'capped' at a rating of '2' for this item.

NB If there is no current mental disorder (broadly defined), the correct rating is zero (0), because the person is not in need of psychiatric admission or follow up.

TU3: TRIAGE URGENCY: SUICIDE PREVENTION

0	No pre-admission assessment **or** no current evidence of mental disorder **or** no suicide risk / behaviour.
1	Low risk currently, low-lethality behaviours.
2	High risk of low-lethality self-harm **or** is already in any hospital placement..
3	High lethality attempts but not recent and not high risk currently (low dynamic risk) while in prison.
4	Recent high lethality suicide attempt, and is in prison. Dynamic risk factors high currently (e.g. recent failed extended suicide or suicide by cop, or stigmatising offence).

Information Quality: □ no information; □ staff observation; □ interview; □ family informants; □ clinical records □ police records □ court papers □ other (tick all informants that apply).

TU4 TRIAGE URGENCY: HUMANITARIAN

This item gives weight to humanitarian and human rights considerations. It is essential to avoid having to impose conditions of treatment or detention that might constitute cruel, unusual or inhuman treatment. If oppressive measures such as physical restraint, seclusion or any other form of coercion are used due to a mental disorder, and if transfer to a therapeutically secure hospital would allow care or treatment without these measures then the transfer should be prioritised accordingly.

Note that in hospital seclusion, restraint and other extraordinary measures may be avoided or minimised by enhanced nursing observations including 2 to 1 nursing and the use of higher staff to patient ratios generally.

NB If there is no current mental disorder (broadly defined), the correct rating is zero (0), because the person is not in need of psychiatric admission or follow up.

TU4 TRIAGE URGENCY: HUMANITARIAN

0	No pre-admission assessment **or** no current evidence of mental disorder **or** no necessity to admit **or** can be managed with precautions but without extra-ordinary means e.g. in a shared cell, with enhanced observation levels.
1	Requires extra-ordinary means in present placement e.g. prolonged seclusion or restraint with no prospect of improvement **and** is in hospital (see TU1D).
2	Is endangering self and others in present place despite extra-ordinary measures e.g. prolonged seclusion or restraint, **but** is currently in a hospital (see TU1D)
3	Requires extra-ordinary means in present placement e.g, prolonged seclusion or restraint with no prospect of improvement **and** is in prison (see TU1B or TU1C)
4	Is endangering self and others in present place despite extra-ordinary measures e.g. prolonged seclusion or restraint, **and** is in prison (see TU1B or TU1C)

Information Quality: □ no information; □ staff observation; □ interview; □ family informants; □ clinical records □ police records □ court papers □ other (tick all informants that apply).

TU 5 TRIAGE URGENCY: SYSTEMIC

This item assesses the extent to which it is systemically appropriate within an overall mental health service for a population, to consider the patient for the level of security provided by this service. A pragmatic, patient centred ‘best interests’ approach must at all times take precedence over other considerations. This is particularly true when catchment area and resource issues are at play in a public health service. As a guide to the appropriateness of admission to a given level of therapeutic security, the DUNDRUM-1 Triage Security rating items and scale should be used.

The distinction made here between ‘soft’ obstacles to admission and ‘hard’ resource issues is an example of pragmatic decision making. Yielding too readily to ‘soft’ obstacles however is systemically dysfunctional and leads to ‘system drift’ whereby appropriately resourced services decline to offer the service for which they have been commissioned and resourced. These issues should wherever possible be resolved by recourse to the DUNDRUM-1 Security items on a case by case basis and as part of a systems audit.

This item may be seen as an additional weighting for issues dealt with in various parts of TU1.

NB If there is no current mental disorder (LEGALLY defined), the correct rating is zero (0), because the person is not in need of psychiatric admission or follow up.

TU 5 TRIAGE URGENCY: SYSTEMIC

0	No mental disorder OR higher levels of therapeutic security are not available.
1	It is necessary to admit the patient to this level of therapeutic security because lower levels though appropriate are not accessible for ‘soft’ reasons e.g. due to catchment area disagreements or local stigma.
2	It is necessary to admit the patient to this level of therapeutic security because a lower level, though appropriate is not available in the catchment area due to resource constraints.<br>NB the more appropriate lower level of therapeutic security should be sought in other catchment areas (see TU1E).
3	Due to assessed triage security needs it is appropriate to admit the patient to this level of therapeutic security because a lower level, though appropriate is not available anywhere in the jurisdiction due to resource constraints.
4	Due to assessed triage security needs, it is appropriate to admit the patient to this level of therapeutic security (not to a lower level) and this is the catchment area service.

Information Quality: □ no information; □ staff observation; □ interview; □ family informants; □ clinical records □ police records □ court papers □ other (tick all informants that apply).

TU6 TRIAGE URGENCY: LEGAL URGENCY

This item give rise to greater conceptual difficulty than any other in this structured professional judgement instrument. All other items reflect the ethical obligation to put the best interests of the person first and to ensure that the appropriate safe therapeutic environment is used to enable the recovery and return to autonomy of the person concerned. This item however prioritises different principles – legal procedures rather than consequences, liberty (in a legal sense) rather than recovery, and where conflicts arise they are often the result of lack of clarification or communication of these issues. N.B. clinical decision makers are advised to seek legal advice as a matter of urgency whenever any difficulty arises in relation to such matters.

It is the view of the authors that legal orders causing the admission of a person who is before the courts in preference to a more medically needy person as rated in these items, particularly DUNDRUM-1 and DUNDRUM-2 are always wrong in principle and in practice. It is the responsibility of the clinicians to ensure that the legal authority making such orders should be aware of the probable consequences of their actions particularly the consequences for those who are for clinical reasons in greater, more urgent need of the hospital bed. There is an inherent injustice when decisions are made deliberately blind to the consequences for others. There is also an inherent error when the responsible decisions normally vested by society in doctors are instead taken by lawyers who are exempt from responsibility for the consequences.

The rating system below prioritises this principle of continuity of responsibility – a decision regarding urgency is more weighty if made by the admitting institution than when made by an expert who carries no clinical responsibility for the consequences.

NB If there is no current mental disorder (LEGALLY defined), the correct rating is zero (0), because the person is not in need of psychiatric admission or follow up.

TU6 TRIAGE URGENCY: LEGAL URGENCY

0	No court order, **or** ‘Order’ to admit by a court that lacks statutory power or inherent powers of High Court (i.e. power to make such an order), or any order that on its face is invalid. NB seek legal advice at once.
1	‘Request’ from any court for a medico-legal report, or for advice or assistance regarding hospital admission – NB an alternative disposal may be more appropriate, see TS items. **Or** ‘Approval’ for admission or transfer by a Mental Health Tribunal.
2	Judicial review or similar proceedings (fitness to stand trial, NGRI, hospital order or restriction order) initiated with a view to admission and likely to succeed. **OR** an order for prison to hospital transfer may be made, subject to bed availability and triage considerations
3	A court order or Mental Health Tribunal order has been made to admit within a defined time period e.g. one or two weeks,<br>**OR** a court order to admit forthwith (JR, Unfit, NGRI) scheduled and likely to be made within the next week.<br>**OR** an order has been made for prison to hospital transfer within a defined time period e.g. one or two weeks
4	A ‘forthwith’ order has been made arising from judicial review or habeas corpus proceedings in connection with detention in prison or elsewhere while awaiting a hospital place.<br>**OR** an order has been correctly completed by a court obliging an admission at once e.g. unfit to plead or NGRI.<br>**OR** a recall order for a conditionally discharged patient has been made and requires admission to this hospital at once

	**OR** an order has been made for prison to hospital transfer with immediate effect.

Information Quality: □ no information; □ staff observation; □ interview; □ family informants; □ clinical records □ police records □ court papers □ other (tick all informants that apply).
